# Sequencing Analysis of Genetic Loci for Resistance for Late Leaf Spot and Rust in Peanut (*Arachis hypogaea* L.)

**DOI:** 10.3389/fpls.2018.01727

**Published:** 2018-11-26

**Authors:** Kenta Shirasawa, Ramesh S. Bhat, Yogendra P. Khedikar, Venkataswamy Sujay, Rohini M. Kolekar, Sharanabasappa B. Yeri, Mallenahally Sukruth, Sarvamangala Cholin, Byregowda Asha, Manish K. Pandey, Rajeev K. Varshney, Makanahally V. C. Gowda

**Affiliations:** ^1^Department of Frontier Research and Development, Kazusa DNA Research Institute (KDRI), Chiba, Japan; ^2^Department of Biotechnology, University of Agricultural Sciences, Dharwad, India; ^3^Department of Genetics and Plant Breeding, University of Agricultural Sciences, Dharwad, India; ^4^Center of Excellence in Genomics and System Biology (CEGSB), International Crops Research Institute for the Semi-Arid Tropics (ICRISAT), Hyderabad, India

**Keywords:** late leaf spot and rust diseases, peanut, quantitative trait locus, restriction-site associated DNA sequencing, whole-genome resequencing analysis

## Abstract

The aim of this study was to identify candidate resistance genes for late leaf spot (LLS) and rust diseases in peanut (*Arachis hypogaea* L.). We used a double-digest restriction-site associated DNA sequencing (ddRAD-Seq) technique based on next-generation sequencing (NGS) for genotyping analysis across the recombinant inbred lines (RILs) derived from a cross between a susceptible line, TAG 24, and a resistant line, GPBD 4. A total of 171 SNPs from the ddRAD-Seq together with 282 markers published in the previous studies were mapped on a genetic map covering 1510.1 cM. Subsequent quantitative trait locus (QTL) analysis revealed major genetic loci for LLS and rust resistance on chromosomes A02 and A03, respectively. Heterogeneous inbred family-derived near isogenic lines and the pedigree of the resistant gene donor, *A. cardenasii* Krapov. & W.C. Greg., including the resistant derivatives of ICGV 86855 and VG 9514 as well as GPBD 4, were employed for whole-genome resequencing analysis. The results indicated the QTL candidates for LLS and rust resistance were located in 1.4- and 2.7-Mb genome regions on A02 and A03, respectively. In these regions, four and six resistance-related genes with deleterious mutations were selected as candidates for LLS and rust resistance, respectively. These delimited genomic regions may be beneficial in breeding programs aimed at improving disease resistance and enhancing peanut productivity.

## Introduction

Peanut (*Arachis hypogaea* L.), so-called groundnut, is an important legume crop widely cultivated for food, oil, and fodder productions. Peanut productivity in most areas is hampered by foliar diseases, particularly late leaf spots (LLS) caused by *Mycosphaerella berkeleyi* W.A. Jenkins, also known as *Phaeoisariopsis personata* (Berk. & M.A. Curtis) van Arx, and rust by *Puccinia arachidis* Speg. These diseases can reduce yield by up to 70% (Subrahmanyam et al., 1984) and can adversely affect kernel and fodder quality (Dwivedi et al., 2002). The production of disease resistant cultivars is limited by genetic bottlenecks, strong associations of disease resistance with poor productivity and undesirable pod characteristics, and inefficient phenotypic selection for resistance, all of which pose challenges for breeding foliar disease resistant varieties. Efficiency of selection can be enhanced using genomics-assisted breeding, which also considerably reduces the time needed for variety development.

The use of molecular breeding techniques is dependent on the availability of trait-linked markers. Efforts have been made to develop linkage maps and identify quantitative trait loci (QTLs) and flanking markers associated with LLS and rust resistance in peanut. The markers flanking these QTL were validated (Yeri et al., 2014; Sukruth et al., 2015) and employed for transferring the LLS and rust resistance regions to susceptible varieties (Varshney et al., 2014; Yeri and Bhat, 2016; Kolekar et al., 2017). Two mapping populations, TAG 24 × GPBD 4 and TG 26 × GPBD 4, were used to construct genetic maps (Khedikar et al., 2010; Sarvamangala et al., 2011; Sujay et al., 2012; Pandey et al., 2014; Kolekar et al., 2016). Initial efforts were concentrated on partial mapping with single sequence repeat (SSR) markers (Khedikar et al., 2010; Sarvamangala et al., 2011; Pandey et al., 2014), followed by map saturation with SSR and transposable element (TE) markers (Sujay et al., 2012; Kolekar et al., 2016). Then, the maps were used for QTL analysis to identify two major genomic regions associated with LLS and rust resistance (Sujay et al., 2012; Kolekar et al., 2016). The first region, on chromosome A03 (linkage group AhXV), governing LLS and rust resistance with very high percentage of variation explained (PVE; up to 82.96%). The second genomic region, on chromosome A02 (linkage group AhXII), contributed to LLS resistance only, with relatively low PVE (62.34%). In addition, another mapping population of VG 9514 × TAG 24 was also employed to detect QTLs for LLS and rust resistance on the A03 (Mondal et al., 2012). There results speculate that the genomic regions on A02 and A03 governing LLS and rust resistance are contributed by *A. cardenasii* Krapov. & W.C. Greg., since the disease resistant parents (GPBD 4 and VG 9514) in the three mapping populations commonly had diploid *A*. *cardenasii* Krapov. & W.C. Greg. in their pedigrees (Bertioli et al., 2016; Pandey et al., 2017).

Genome sequences are now available for the A- and B-genome diploid progenitors of peanut, namely *A. duranensis* Krapov. & W.C. Greg. and *A. ipaeënsis* Krapov. & W.C. Greg., respectively (Bertioli et al., 2016). As a result, mapping efforts are currently focused on genome sequence-based analysis (Pandey et al., 2017; Clevenger et al., 2018) and single-nucleotide polymorphism (SNP)-enriched mapping to delimit regions of interest and identify candidate genes. Pandey et al. (2017) reported 25 candidate genes for LLS resistance and nine candidates for rust on a 3.06-Mb region of the A03 by a QTL-Seq approach, in which bulks of resistance and susceptible lines of the TAG 24 × GPBD 4 population were employed. Recently, Mondal and Badigannavar (2018) have also identified five candidate resistance genes for rust on A03 using the gene function prediction/annotation data and domain search analysis.

In this study an effort was made to map the genomic regions governing LLS and rust resistance using an improved genetic map and extensive phenotypic data. To dissect the A02 and A03 chromosomal regions using genetic mapping, 190 SNPs newly developed in this study were added to the previously reported map of TAG 24 × GPBD 4 with the 263 published markers (Khedikar et al., 2010; Sarvamangala et al., 2011; Sujay et al., 2012). The updated high-density map was employed for high-resolution QTL mapping, in which phenotypic data for the LLS and rust across 11 seasons (2004–2014) were used. In addition, the chromosomal regions were validated for LLS and rust resistance by comparing SNPs from WGRS data in bulked samples of heterogeneous inbred family (HIF)-derived near isogenic lines (NILs), that genetic background is different from the RILs of TAG 24 × GPBD 4 as Pandey et al. (2017) were used for the QTL-Seq analysis. Also, co-segregation between the disease resistance phenotypes and genomic regions was examined by comparing SNPs from whole-genome resequencing (WGRS) data between resistant (GPBD 4, ICGV 86855, and VG 9514) and susceptible (TAG 24 and five Japanese lines) genotypes. This study integrated genetic and genomic analysis to identify the genic SNPs on A02 and A03 that co-segregated with LLS and rust resistance.

## Materials and Methods

### Plant Materials

The pedigree of the plant materials used in this study is shown in Supplementary Figure S1 and Supplementary Table S1. A TAG 24 × GPBD 4 RIL mapping population consisting of 266 RILs was used for QTL analysis. This mapping population was developed previously by crossing an LLS and rust susceptible variety, TAG 24 (Patil et al., 1995), with a resistant variety, GPBD 4 (Gowda et al., 2002), and advancing the generations by single seed decent (SSD) (Khedikar et al., 2010; Sarvamangala et al., 2011; Sujay et al., 2012). In addition, a total of 29 heterogeneous inbred family (HIF)-derived near isogenic lines (NILs) (Yeri et al., 2014), derived from crosses between TAG 24 and GPBD 4, and between another susceptible variety, TG 26 (Kale et al., 1997), and GPBD 4, were used for bulked segregant analysis by sequencing. The HIF-NILs consisted of 13 rust-resistant (1-1, 7-2, 9-2, 14-1, 50-2, 60-3, 77-1, 83-1, 89-1, 46-3, 53-1, 101-1, and 116-1) and 16 rust-susceptible (1-2, 7-1, 9-1, 9-3, 14-2, 46-1, 46-2, 50-1, 53-2, 60-1, 60-2, 77-2, 83-2, 89-2, 101-2, and 116-2) lines. The phenotyping scores of the resistance lines and susceptible were reported to be 4.55 (4.13–4.88) and 6.41 (5.38–7.75) on average, respectively (Yeri et al., 2014), where susceptible and resistance should have scores of >5 and <5.

### SNP Genotyping by ddRAD-Seq Analysis

Genome-wide SNP analysis across the RIL population was conducted using ddRAD-Seq (Peterson et al., 2012). Genomic DNAs extracted from leaves of each RIL and their parental lines with DNeasy Plant Mini Kit (Qiagen, Germany) were double-digested with the restriction enzymes *Pst*I and *Msp*I (Thermo Fisher Scientific, MA, United States). The ddRAD-Seq libraries were constructed as described by Shirasawa et al. (2016a,b). The libraries were sequenced on HiSeq2000 (Illumina) in paired-end mode (93-base). Nucleotide sequence data were deposited in the DDBJ Sequence Read Archive (accession numbers DRA006500, DRA006514, and DRA006515).

Data processing including quality control of raw sequence reads, adaptor trimming, mapping of trimmed reads onto reference sequences, and SNP calling, was performed as described by Shirasawa et al. (2016a,b). Low-quality sequences were removed and adapters were trimmed using PRINSEQ and fastx_clipper in FASTX-Toolkit^1^. The filtered reads were mapped onto a genome sequence concatenated. from two diploid progenitorS of peanut, *A. duranensis* Krapov. & W.C. Greg. and *A. ipaënsis* Krapov. & W.C. Greg. (Bertioli et al., 2016), as a reference using Bowtie 2 (Langmead and Salzberg, 2012). The resultant sequence alignment–map format (SAM) files were converted to binary sequence alignment–map format (BAM) files and subjected to SNP calling using the mpileup option of SAMtools and the view option of BCFtools (Li et al., 2009). Lengths of genome regions covered with at least two reads were calculated with the genome Coverage option of BEDtools (Quinlan and Hall, 2010). High-confidence biallelic SNP candidates were selected using VCFtools (version 0.1.12b: Danecek et al., 2011) with the following criteria: (i) depth of coverage ≥5 for each data point, (ii) SNP quality score of ≥20 for each locus, and (iii) proportion of missing data of <50% for each locus.

### Linkage Map Construction and QTL Analysis

Linkage analysis was performed for the TAG 24 × GPBD 4 population with JoinMap 4.0 (Van Ooijen, 2006). The “Locus Genotype Frequency” function was used to calculate Chi-square (χ^2^) values for each marker to test for the expected 1:1 segregation ratio Markers were placed into linkage groups with the “LOD Groupings” and “Create Groups for Mapping” command using the Kosambi mapping function (Kosambi, 1943). Calculation parameters were set for a minimum LOD (logarithm of the odds) of 3 and recombination fraction of 0.45. Marker order in groups was established using the “Calculate Map” command. The linkage map was constructed using MapChart 2.2 software (Voorrips, 2002).

Detection of QTL associated with responses to LLS and rust among the TAG 24 × GPBD 4 population was performed using a composite interval mapping (CIM) approach (Zeng, 1994) using WinQTL Cartographer, version 2.5 (Wang et al., 2011). CIM was performed using Model 6, with scanning intervals of 2.0 cM between markers and putative QTL with a window size of 10.0 cM. The number of marker cofactors for the background control was set by forward–backward stepwise regression. The “Locate QTLs” option was used automatically with a minimum of 5 cM between QTL in order to define a QTL region. One thousand permutationsaq were employed for determining the QTL using the option “permutations times” with 0.05 significance level.

### Bulked Segregant Analysis by Sequencing

For bulked segregant analysis by sequencing, genomic DNAs from the 13 resistant and 16 susceptible lines of the HIF-NILs (Yeri et al., 2014) were mixed into two pools (resistant and susceptible) and subjected to library preparation (paired-end libraries with insert size of 500 bp) as described by Shirasawa et al. (2016b). The libraries were sequenced on NextSeq 500 systems (Illumina) in paired-end mode (151-base). Nucleotide sequence data were deposited in the DDBJ Sequence Read Archive (accession number DRA006500). SNP calling was modified as follows. BAM files were subjected to SNP calling using the mpileup option of SAMtools (version 0.1.19) and the mpileup2snp option of VarScan 2 (version 2.3: Koboldt et al., 2012) to obtain a variant call format (VCF) file including SNP information. High-confidence biallelic SNP candidates were selected using VCFtools (version 0.1.12b: Danecek et al., 2011) with the following criteria: (i) depth of coverage ≥10 for each data point and (ii) no missing data for each locus. The effects of SNPs on gene function were predicted using SnpEff v4.1g (Cingolani et al., 2012). From the filtered VCF files, values for variant allele frequency (FREQ) for each position were extracted from genotype fields. FREQ values over the genome were plotted using R (R Core Team, 2013). Genetic loci significantly associated with SNPs (*P* < 0.01) were selected under the null hypothesis of no QTLs as reported by Takagi et al. (2013) and Pandey et al. (2017).

### Whole Genome Resequencing Analysis

WGRS data for ICGV 86855 and VG 9514 (resistant lines) and Chiba-handachi, Satonoka, Kintoki, Nakateyutaka, and YI-0311 (susceptible lines) were obtained from the DNA Data Bank of Japan (DDBJ) Sequence Read Archive (Shirasawa et al., 2016b; Gayathri et al., 2018; accession numbers DRA004503 and DRA006239 ^2^). Data for TAG 24 (a susceptible line) and GPBD 4 (a resistant line) were provided by The International Crops Research Institute for the Semi-Arid Tropics (Pandey et al., 2017). Data processing of WGRS reads was performed as above except for SNP filtering. High-confidence biallelic SNP candidates were selected using VCFtools (version 0.1.12b: Danecek et al., 2011) with the following criteria: (i) depth of coverage ≥5 for each data point, (ii) SNP quality score of ≥999 for each locus, and (iii) no missing data for each locus. The effects of SNPs on gene function were predicted using SnpEff v4.1g (Cingolani et al., 2012).

## Results

### QTL Analysis of Rust and LLS Resistance

An average of 1.7 million (M) high-quality ddRAD-Seq reads per sample were obtained from a TAG 24 × GPBD 4 RIL population (Supplementary Table S2). Reads were mapped onto genome reference sequences of *A. duranensis* Krapov. & W.C. Greg. and *A. ipaënsis* Krapov. & W.C. Greg., with an average map rate of 85.4% for TAG 24 × GPBD 4. Approximately 3.2 Mb (0.13%) of the genome was covered with at least five reads. From the read alignments, 190 SNPs were detected with high confidence. The SNPs were named as a combination of names of chromosomes and positions by linking with an underscore. These SNPs together with the 326 SSR (Sujay et al., 2012) and *A. hypogaea* L. transposable element (AhTE) (Kolekar et al., 2016) markers were used for a linkage analysis. The resultant map consisted of 29 linkage groups with 453 loci (171 SNPs, 89 transposons, and 193 SSRs) covering a total length of 1510.1 cM (Supplementary Table S3 and Supplementary Figure S2). Clusters of SNPs were observed in AhXIIb (A02) and AhXV (A03). In accordance with the SNPs, 23 linkage groups were assigned to 18 chromosomes expect for A07 and A10, while the other six groups had no SNP loci. Among the 171 mapped SNPs, only three were unexpectedly located on different chromosomes (but homoeologous chromosomes), e.g., Aradu.A06_106278061 and Aradu.A06_108579782 were on AhX (B06) and Araip.B03_125319254 was on AhIIIa (A03).

This genetic map was used for QTL analysis using the phenotypic data on the LLS and rust reaction collected over 11 seasons (2004–2014) (Khedikar et al., 2010; Sujay et al., 2012; Kolekar et al., 2016). The phenotypic data has suggested high heritability and independent nature of inheritance between both the diseases (Khedikar et al., 2010). For LLS resistance, QTLs were detected on 12 linkage groups (Supplementary Table S4 and Supplementary Figure S2). Of these, two QTL regions with high LOD/PVE were detected on AhXIIb (A02) and AhXV (A03). The QTL on A02 were stable across eight seasons with a maximum additive effect 1.81 for the favorable allele contributed from GPBD 4, and PVE of up to 91.51%. For rust resistance, a single QTL region was found on AhXV (A03) with a maximum PVE of 35.72% and the additive effect of 2.06 for the favorable allele of GPBD 4 (Supplementary Table S4 and Supplementary Figure S2). The genotypes of the markers on the QTLs were highly associated with the phenotypes (Supplementary Figure S3).

### Identification of Genome Fragment Conferring Rust Disease Resistance

**FIGURE 1:**
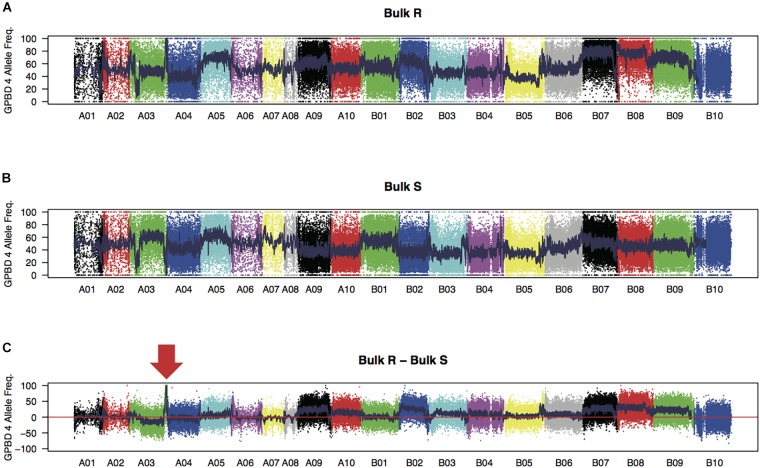
SNP signals detected by bulked segregant analysis by sequencing. Frequencies of GPBD 4 alleles of genome-wide SNPs in bulk samples of rust-disease susceptible lines, bulk S **(A)**, and those of resistant lines, bulk R **(B)**, are plotted. Differences between the bulks R and S (bulk R – bulk S) are shown in **(C)** with an arrow indicating a prominent peak at the end of the chromosome A03. Twenty chromosomes were painted by different colors.

WGRS analysis produced 315 and 319 million high-quality 151 nucleotide reads from the bulk samples of resistant and susceptible lines, respectively (Supplementary Table S2). Reads were mapped onto the reference sequence with an average alignment rate of 94.0%. The sequence reads for each sample covered approximately 1.7 Gb of the genomic regions (72.0% of the reference sequences) with at least 10 reads. Average sequence read depth was 28× for each sample. A total of 173,995 high-quality SNPs, with 67,251 and 106,744 SNPs on the A and B genomes, respectively, were detected between the two bulk samples. The expected FREQ values of the GPBD 4 alleles in the resistant and susceptible bulk-sample were 1 and 0, respectively, at the resistance loci. These predicted values were observed in a 5.2 Mb region of chromosome A03 (position 129.7–134.9 Mb) with a significant level (*P* < 0.01) (Figure 1), suggesting that the candidate loci for rust resistance were located in this region.

**FIGURE 2:**
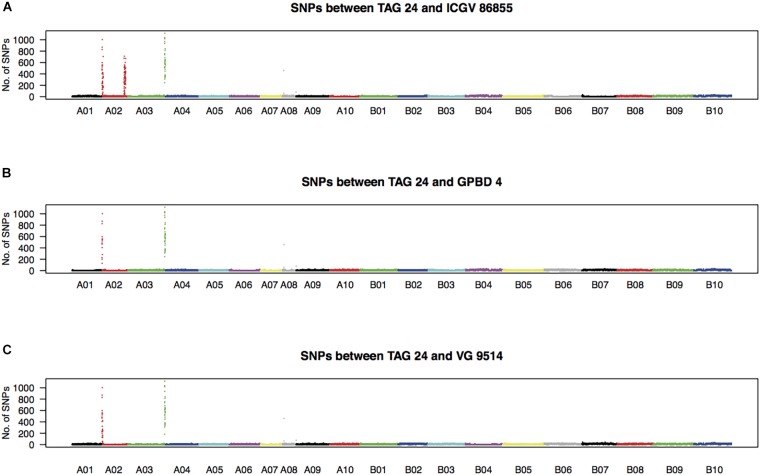
Density of SNPs in resistant lines with respect to TAG 24. Numbers of SNPs every 100-kb length over the genome between TAG 24 and either ICGV 86855 **(A)**, GPBD 4 **(B)**, or VG 9514 **(C)** are plotted.

**FIGURE 3:**
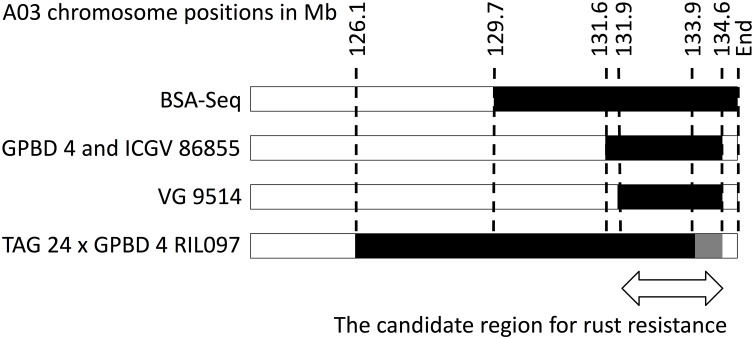
Graphical genotypes of A03 for resistant lines. Black bars show chromosome segments derived from the resistant donor, GPBD 4. Numbers on the top are chromosome positions in a mega-base scale. An arrow indicates an overlapping region of the GPDB 4 genome corresponding to the candidate region for rust resistance.

High-quality WGRS reads, approximately 20–40× genome coverage, were produced for the three resistant (GPBD 4, ICGV 86855, and VG 9514) and six susceptible (TAG 24, Chiba-handachi, Nakateyutaka, YI-0311, Satonoka, and Kintoki) lines and were mapped onto the reference sequence with an alignment rate of 96.1% (Supplementary Table S2). The reads covered 2.0 Gb (83.8%) of the genome with at least five reads. From the alignment data, 3,532,384 and 168,116 SNPs between the tested lines and the reference sequences were detected on the A and B genomes, respectively. Conversely, 189,559 A-genome SNPs and 252,983 B-genome SNPs were found among the tested lines. With respect to TAG 24, there were three SNP clusters in ICGV 86855 (0–4.5 Mb on A02; 72.9–85.9 Mb on A02; and 131.6–134.6 Mb on A03), two SNP clusters in GPBD 4 (0–1.4 Mb on A02 and 131.6–134.6 Mb on A03), and two SNP clusters in VG 9514 (0–3.1 Mb on A02 and 131.9–134.6 Mb on A03) (Figure 2). No prominent signals were observed in the other five susceptible lines (Supplementary Figure S4). The positions of the SNP “hot-spots” in the resistance lines overlapped, indicating that the three resistant lines, descendants of *A. cardenasii* Krapov. & W.C. Greg. (GKP10017, PI262141), shared the same haplotypes in these regions. Subsequently, the candidate region for rust resistance was refined to an approximately 2.7 Mb genome fragment (131.9–134.6 Mb on A03) (Figure 3). The region contributing for LLS resistance was found at 0–1.4 Mb on A02.

### Potential Candidate Genes for Rust and LLS Resistance

The 1.4 Mb candidate region for LLS on A02 included 147 predicted genes (Supplementary Table S5). Of these, nine genes with “leucine-rich repeat (LRR)” and “mildew resistance locus O (MLO)” annotations were considered to be candidates for LLS resistance. Three of these nine genes (Aradu.0G2IC, Aradu.M8RMT, and Aradu.F918E) had sequence variations in the coding regions that caused nonsense- or missense mutations (Table 1). Nonsense mutations were seen across susceptible-line specific alleles of Aradu.0G2IC (encoding LRR and NB-ARC domain disease resistance proteins) and in resistance-line alleles of Aradu.M8RMT (disease resistance protein). Aradu.F918E (LRR and NB-ARC domain disease resistance protein) had a missense mutation at amino acid 66 that produced glutamate and lysine, respectively, in the alleles of the susceptible and resistance lines (hereafter termed Glu^R^66Lys^S^, with superscripts indicating alleles of resistant (R) and susceptible (S) lines).

**Table 1:** Candidate genes for LLS and rust resistance.

Gene ID	Chromosome	Gene position		Annotation	Amino-acid sequence mutation
		Start	End		
**LLS resistance candidates**					
Aradu.0G2IC	Aradu.A02	425,739	442,634	LRR and NB-ARC domain disease resistance protein	Gln599^∗^
Aradu.M8RMT	Aradu.A02	442,691	475,217	Disease resistance protein (TIR-NBS-LRR class)	Tyr574^∗^
Aradu.5Y217	Aradu.A02	1,393,757	1,400,118	MLO-like protein 1-like [Glycine max]	No mutations
Aradu.F918E	Aradu.A02	1,419,387	1,422,860	LRR and NB-ARC domain disease resistance protein	Glu66Lys
**Rust resistance candidates**					
Aradu.C88Z1	Aradu.A03	133,033,579	133,038,386	Seed linoleate 9S-lipoxygenase	His307Arg, Gly49Arg, and Leu34Ser
Aradu.Z87JB	Aradu.A03	133,776,796	133,780,539	Disease resistance protein (TIR-NBS-LRR class), putative	Ile27Val
Aradu.1WV86	Aradu.A03	133,878,019	133,879,319	Glucan endo-1,3-beta-glucosidase-like protein 2-like [Glycine max]	Cys8Tyr
Aradu.RW91L	Aradu.A03	133,933,250	133,935,646	Lipase/lipooxygenase, PLAT/LH2 family protein	Glu344Ala
Aradu.NG5IQ	Aradu.A03	133,995,919	133,999,850	Glucan endo-1,3-beta-glucosidase 4-like [Glycine max]	Lys127Glu, Pro116Leu, Ser72Cys, and Gly69Cys
Aradu.YL3ZN	Aradu.A03	134,333,421	134,335,845	Receptor-like kinase 1	Arg47Ser


*^∗^Stop codons.*

The 2.7 Mb region of A03 associated with rust resistance contained 221 genes (Supplementary Table S6). Seven candidate resistance genes were selected using the annotation terms “leucine-rich repeat (LRR),” “beta-glucan,” “lipoxygenase (LOX),” and “two-component system.” Of these, while no deleterious mutation was found in one gene, 11 missense variations were predicted in the remaining six genes (Table 1): Ile^S^27Val^R^ in Aradu.Z87JB [putative disease resistance protein (TIR-NBS-LRR class)]; His^S^307Arg^R^, Gly^R^49Arg^S^, and Leu^R^34Ser^S^ in Aradu.C88Z1 (seed linoleate 9S-lipoxygenase); Cys^R^8Tyr^S^ in Aradu.1WV86 (glucan endo-1,3-beta-glucosidase-like protein 2-like); Glu^S^344Ala^R^ in Aradu.RW91L (Lipase/lipooxygenase, PLAT/LH2 family protein); Lys^S^127Glu^R^, Pro^R^116Leu^S^, Ser^S^72Cys^R^, and Gly^R^69Cys^S^ in Aradu.NG5IQ (glucan endo-1,3-beta-glucosidase 4-like); and Arg^R^47Ser^S^ in Aradu.YL3ZN (receptor-like kinase 1).

## Discussion

There were three points that we achieved in this study. The first is establishment of a high-density genetic map of TAG 24 × GPBD 4 (Supplementary Table S3 and Supplementary Figure S2) by employing 171 SNPs newly developed from this study, for which the ddRAD-Seq technology was indeed helpful. The number of the SNPs was lower and higher than those detected by Zhou et al. (2014) and Shirasawa et al. (2016b), respectively. This might due to differences of genetic diversity of the tested lines as reported by Shirasawa et al. (2016a), as well as data processing conditions such as criteria for filtering low quality SNPs. Out of the 171 SNPs, three (1.8%) were assigned to unexpected linkage groups, homoeologous chromosomes, as reported by Agarwa et al. (2018) where 739 of 8869 SNPs (8.3%) were mis-assigned due to the similarity of the sequences between the A and B genomes. Totally, however, this mapping result contributed to increase the number of markers on the map up to 1.5 times rather than the previous version with 289 marker loci (Kolekar et al., 2016), and marker density was also improved from 6.0 cM/locus (Kolekar et al., 2016) to 3.3 cM/locus in this study. Subsequent QTL analysis indicated the results of the previous studies (Khedikar et al., 2010; Sarvamangala et al., 2011; Sujay et al., 2012) were reproducible and promised the SNPs clustered in the QTL regions could be a source for DNA markers to select LLS and rust resistance lines in future breeding programs (Supplementary Figure S3).

Second point is that, to the best of our knowledge, this study is the first detailed genomic exploration of the QTLs on A02 and A03. NGS-based strategies were used to refine the candidate regions to a 1.4 Mb genome segment of A02 for LLS resistance and a 2.7 Mb segment of A03 for rust resistance (Figure 3 and Supplementary Table S5, S6). These segment sizes were shorter than those reported by Pandey et al. (2017), suggesting that it would be effective to use materials with novel genetic background that are expected to have new recombination breakpoints as in VG 9514 (Supplementary Table S1 and Supplementary Figure S1). Furthermore, WGRS analysis indicated that there were SNP hotspots in the QTL regions (Figure 2), that haplotype was conserved in the three resistant lines, ICGV 86855, GPBD 4, and VG 9514. Whereas we postulated that these regions might be derived from *A. cardenasii* Krapov. & W.C. Greg. in accordance with the pedigree (Supplementary Table S1 and Supplementary Figure S1) and the result of IntroMap (Clevenger et al., 2017), further study providing direct experimental evidences are required to confirm our speculation.

In addition, as third point, WGRS data supported the presumed effects of SNPs on gene function, which information was helpful to identify four and six candidate genes for LLS and rust resistance, respectively (Table 1). Certainly, gene annotation terms were also useful to delimit the candidates as reported by Pandey et al. (2017) and Mondal and Badigannavar (2018). Out of the candidates, three genes (Aradu.Z87JB, Aradu.1WV86, and Aradu.NG5IQ) were overlapped those suggested in the previous studies (Pandey et al., 2017; Mondal and Badigannavar, 2018). Obviously, advanced genetic analysis is required to identify the responsible sequence genes/variations for rust resistance. Because we identified a single RIL with a recombination breakpoint in the A03 candidate region (Figure 3), progeny tests of this line would be one possibility to further delimit the candidate region.

## Conclusion

In conclusion, we demonstrated integration of genetic and physical mapping could facilitate identification and validation of genomic regions governing traits, and use of genotypes with different genomic backgrounds would assist in rapid identification of candidate genes. However, the physical mapping in this study was based on the reference genome sequences of two wild diploids, *A. duranensis* Krapov. & W.C. Greg. and *A. ipaensis* Krapov. & W.C. Greg. Once the genome sequence of the cultivated peanut is disclosed and used for physical mapping, any discrepancies due to chromosome-level structural variations such as translocations, inversions, copy number variations, and present/absent variations between the wild and cultivated genome sequences can be resolved. The expected results from the cultivated peanut genome as well as the current knowledge based on the wild progenitor genomes would prove valuable for transferring resistance to LLS and rust to elite varieties to enhance peanut productivity.

## Author Contributions

KS and RSB conceived the study and designed the experiments. MVCG developed and provided the mapping population and GPBD 4. YPK, SC, VS, RMK, BA, and SBY conducted the phenotyping. MS made the genetic map and performed the QTL mapping. KS produced and processed the NGS data. MKP and RKV provided the sequence data for TAG 24 and GPBD 4. KS and RSB analyzed and interpreted the data and wrote the manuscript. All authors read and approved the final manuscript.

## Conflict of Interest Statement

The authors declare that the research was conducted in the absence of any commercial or financial relationships that could be construed as a potential conflict of interest.

## Footnotes

1 http://hannonlab.cshl.edu/fastx_toolkit

2 http://www.ddbj.nig.ac.jp
