# Supplementary material for: Sequencing Analysis of Genetic Loci for Resistance for Late Leaf Spot and Rust in Peanut (Arachis hypogaea L.)
Source: Front Plant Sci. 2018 Nov 26;9:1727. doi: 10.3389/fpls.2018.01727 (PMC6275244; doi:10.3389/fpls.2018.01727)
Supplement: Supplementary file 1 [file Data_Sheet_1.PDF]

Supplementary information

Sequencing analysis of genetic loci for resistance for late leaf spot and rust in peanut (*Arachis hypogaea* L.)

Kenta Shirasawa[1]*, Ramesh S. Bhat[2], Yogendra P. Khedikar[3], Venkataswamy Sujay[3], Rohini M. Kolekar[2], Sharanabasappa B. Yeri[2], Mallenahally Sukruth[2], Sarvamangala Cholin[3], Byregowda Asha[2], Manish K. Pandey[4], Rajeev K. Varshney[4], and Makanahally V. C. Gowda[3]

[1]Department of Frontier Research and Development, Kazusa DNA Research Institute (KDRI), Chiba 292-0818, Japan; [2]Department of Biotechnology, University of Agricultural Sciences, Dharwad 580 005, India; [3]Department of Genetics and Plant Breeding, University of Agricultural Sciences, Dharwad 580 005, India; and [4]Center of Excellence in Genomics and System Biology (CEGSB), International Crops Research Institute for the Semi-Arid Tropics (ICRISAT), Hyderabad 502 324, India

*Correspondence:
Kenta Shirasawa
shirasaw@kazusa.or.jp

Supplementary Table S1. Description of plant materials used in this study and their pedigree

Supplementary Table S2. Number of sequence reads and aligned rate on the reference sequence

Supplementary Table S3. Details of the genetic map based on the RILs of TAG 24 × GPDB 4

Supplementary Table S4. QTLs for LLS and rust resistances detected in the TAG 24 × GPDB 4 RIL population

Supplementary Table S5. Gene list in the candidate regions for LLS resistance

Supplementary Table S6. Gene list in the candidate regions for rust resistance

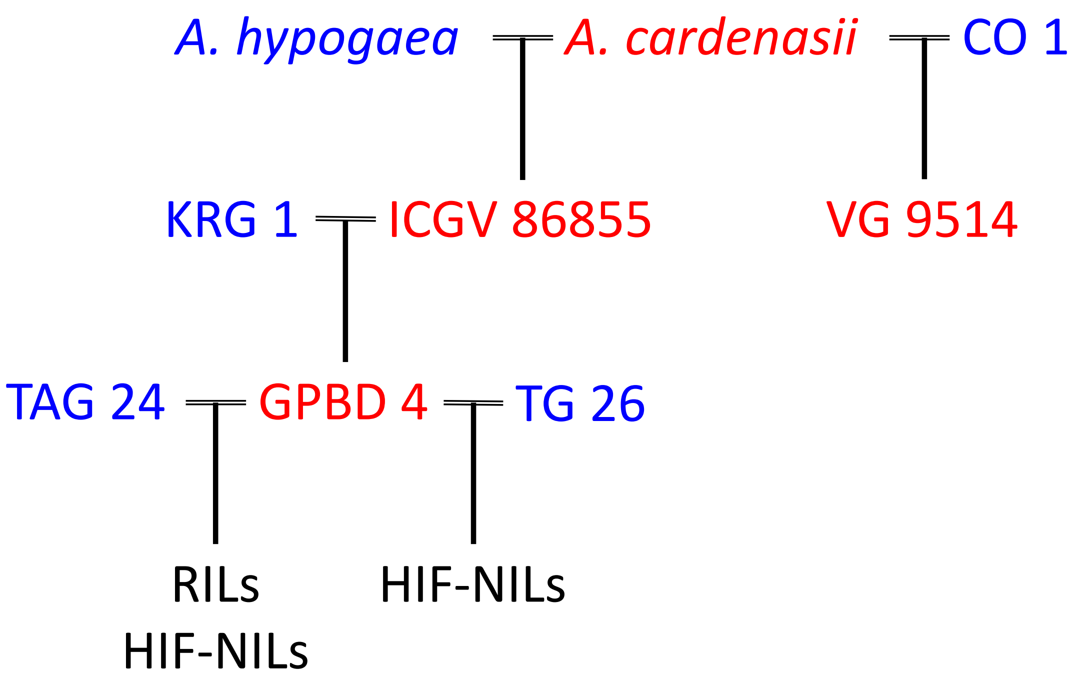

Supplementary Fig S1. The pedigree of the plant materials

Lines in red letters are resistance to LLS and rust, while those in blue are susceptible.

Details of the materials are described in Supplementary Table S1.

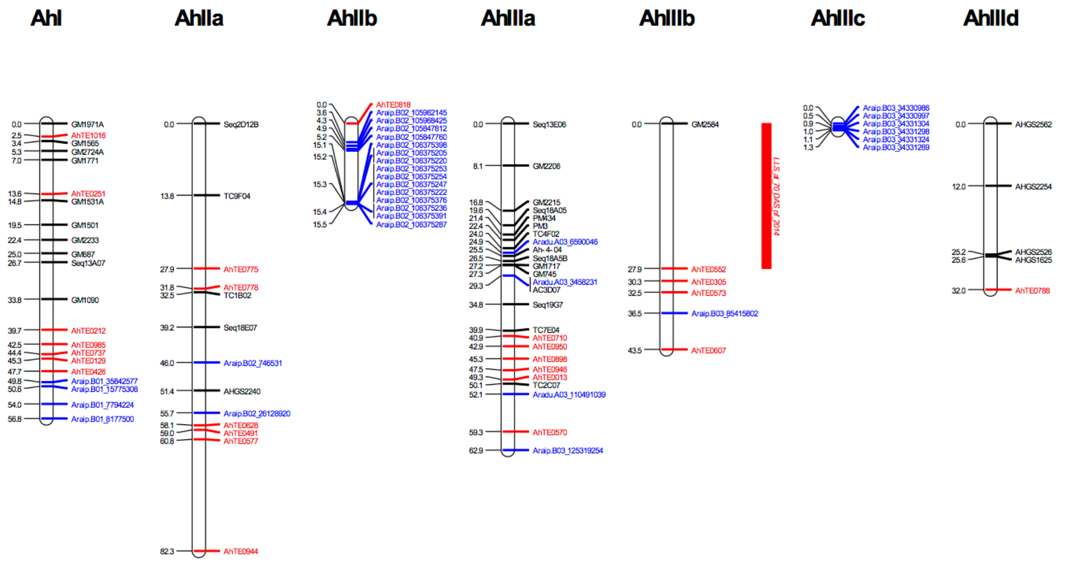

Supplementary Fig S2. The genetic map based on the TAG 24 × GPDB 4 RIL population and the QTL positions for rust and LLS resistances

Genetic loci of SSR, AhTE, and SNP markers are indicated in black, red, and blue, respectively. QTLs for rust and LLS resistances are indicated by blue and red bars, respectively.

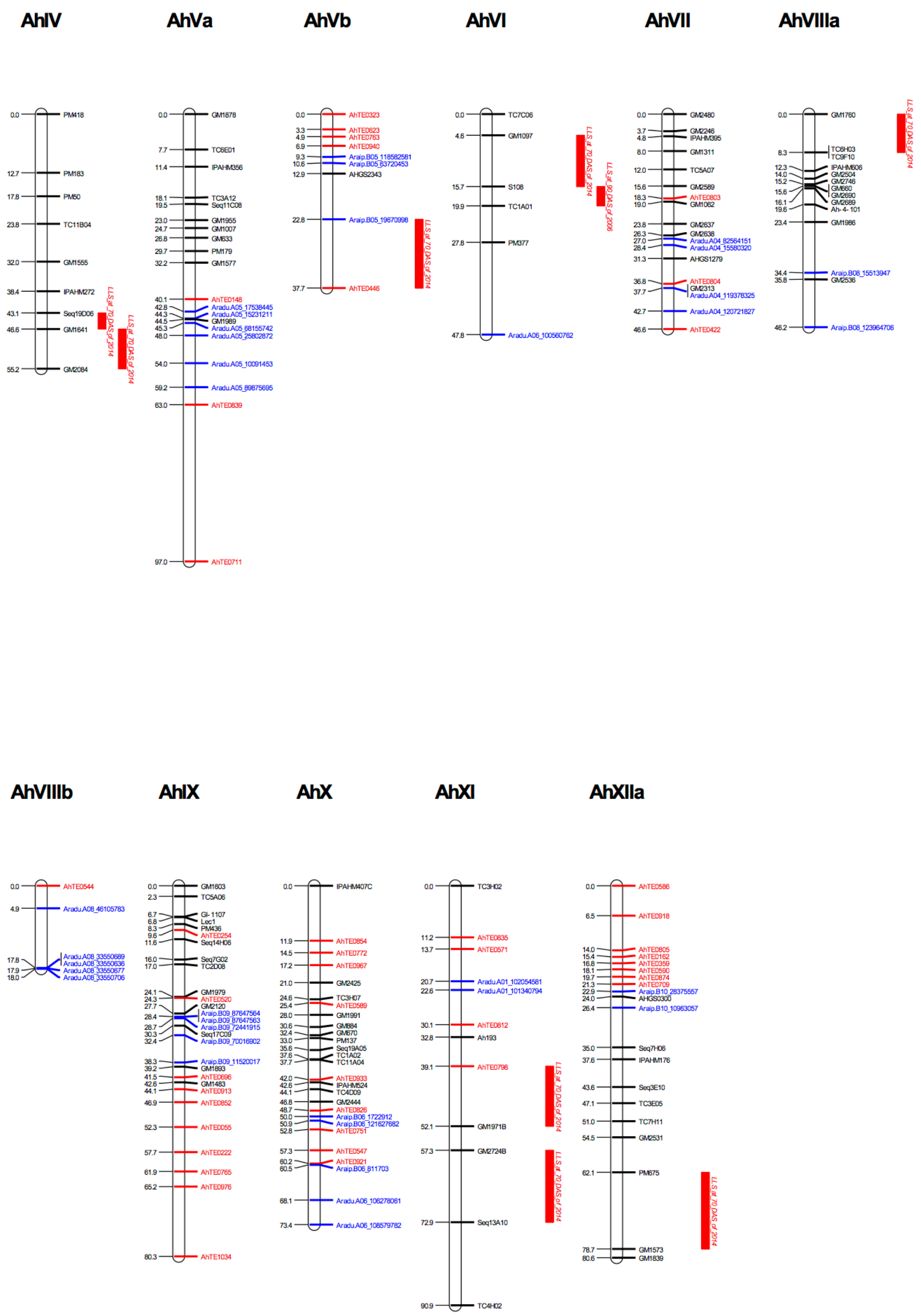

Supplementary Fig S2. (continued)

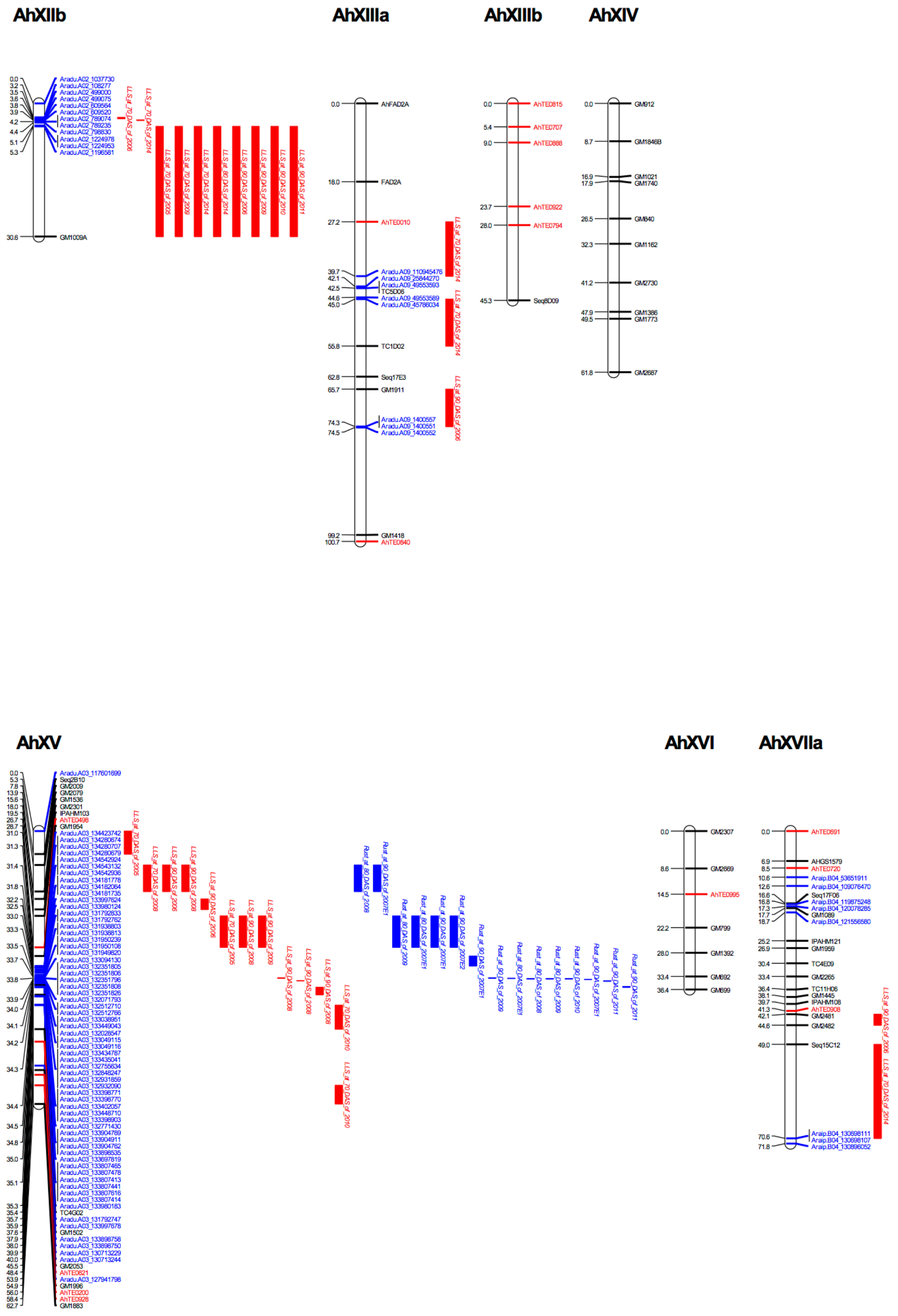

Supplementary Fig S2. (continued)

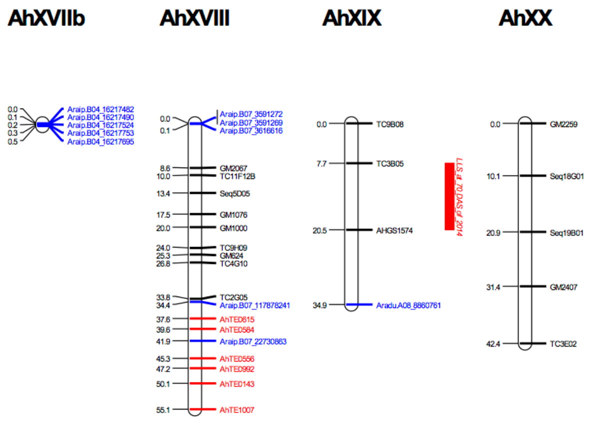

Supplementary Fig S2. (continued)

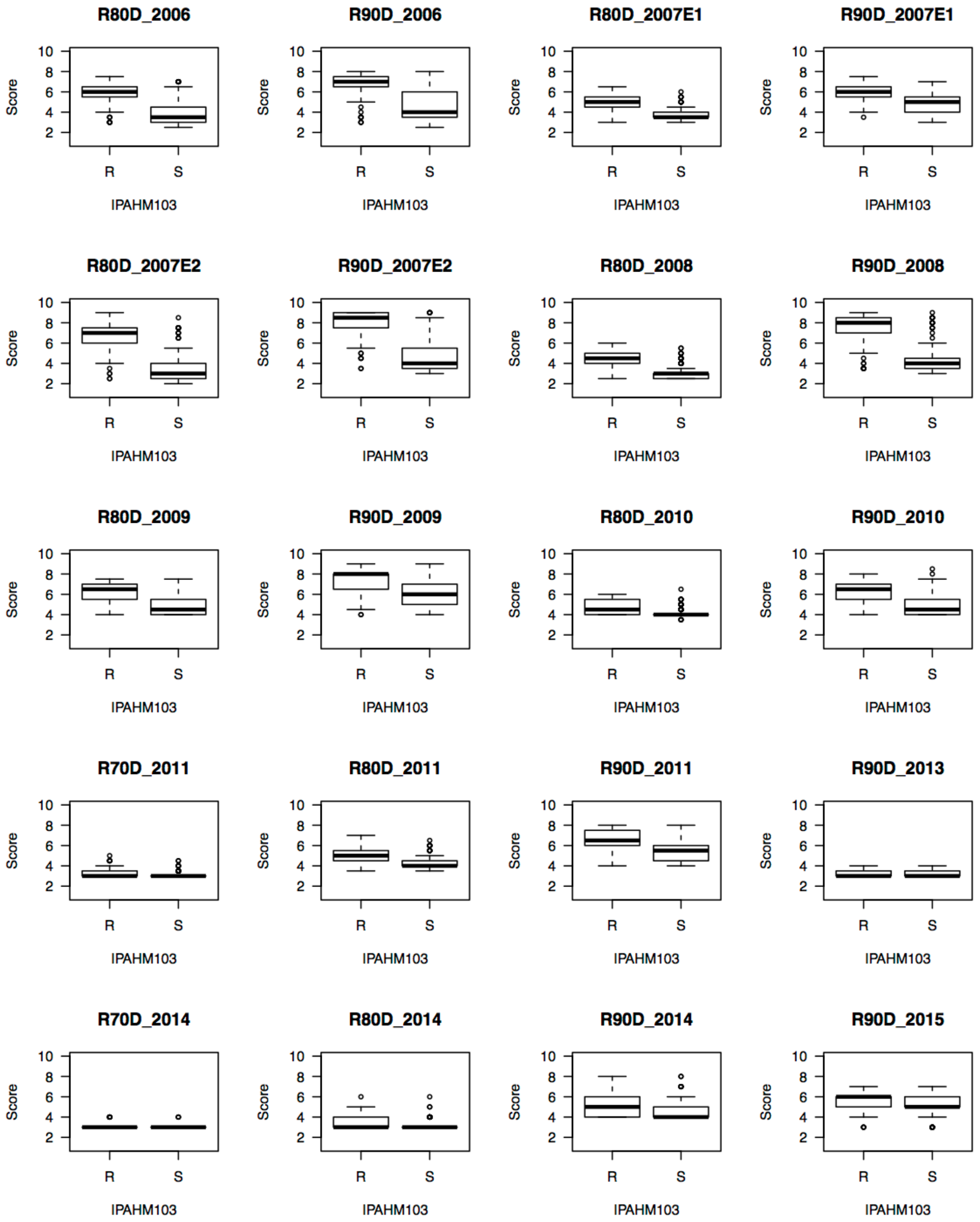

Supplementary Fig S3. Phenotype scores for rust and LLS resistances based on the genotypes of the markers in QTL regions

Aradu.A02_1196581 on A02 and IPAHM103 on A03 are representative markers for rust and LLS resistances. The allele of GPBD 4 (resistant) and TAG 24 (susceptible) are indicated with R and S, respectively.

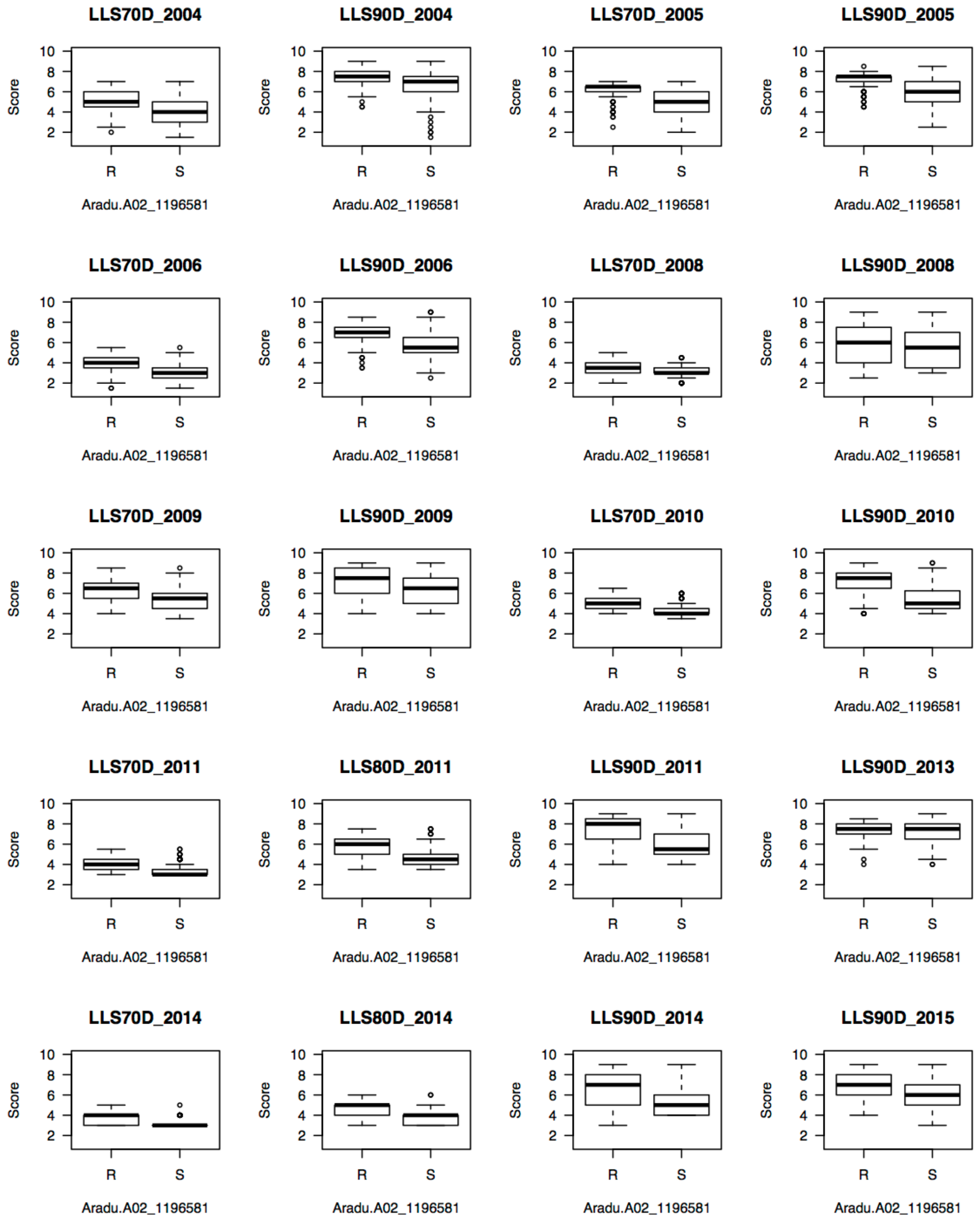

Supplementary Fig S3. (continued)

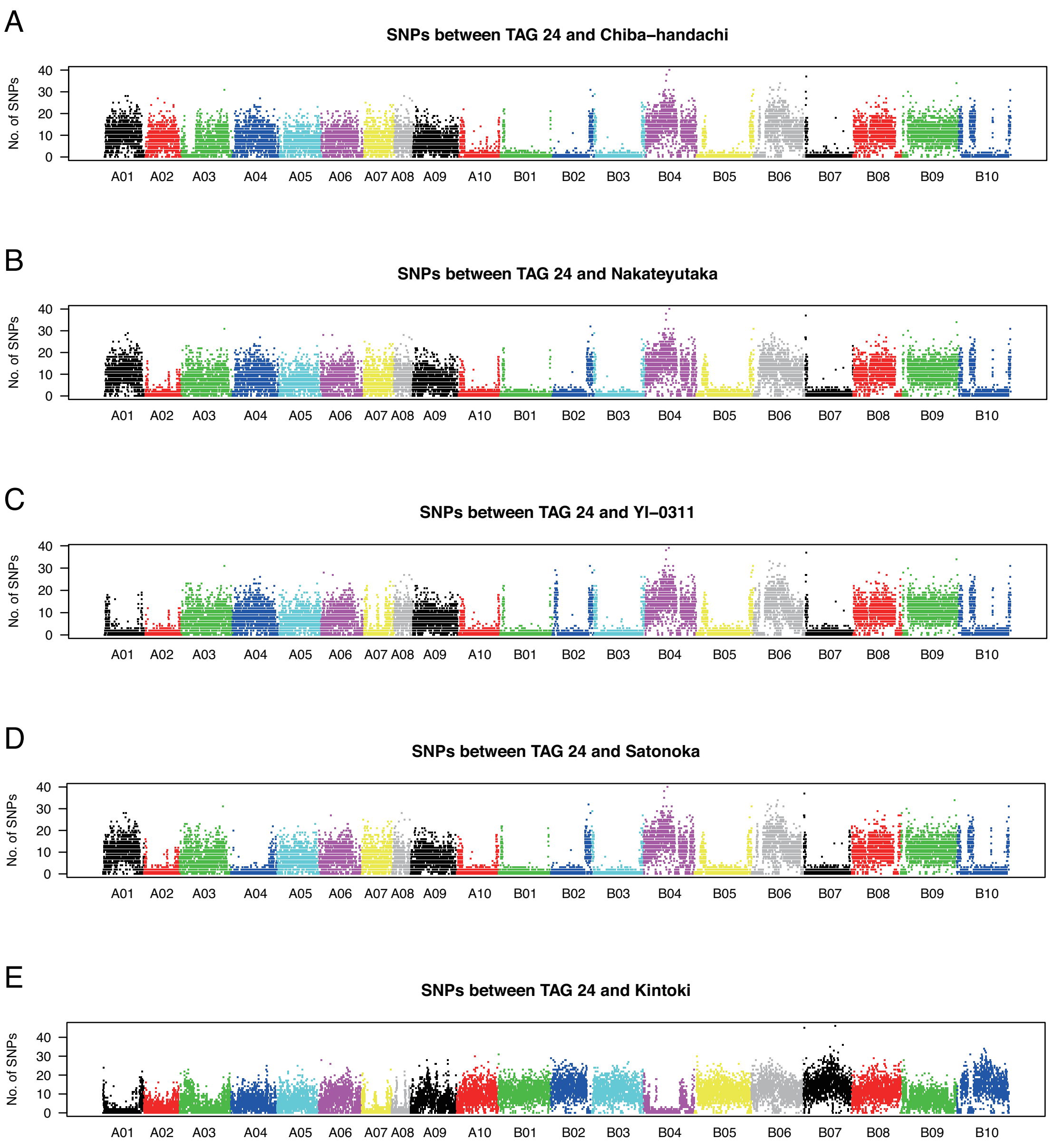

Supplementary Fig S4. Density of SNPs in susceptible lines with respect to TAG 24 Numbers of SNPs every 100-kb length over the genome between TAG 24 and either Chiba-handachi (A), Nakateyutaka (B), YI-0311 (C), Satonoka (D), and Kintoki (E) are plotted.
